# How Non-Essentialism Beliefs of Aging Shapes Awareness of Age-Related Changes: a Cross-Cultural Perspective

**DOI:** 10.1093/geroni/igaf122.2202

**Published:** 2025-12-31

**Authors:** Yiwen Wu, Helene Fung

**Affiliations:** The Chinese University of Hong Kong, Hong Kong, China (People’s Republic); The Chinese University of Hong Kong, Hong Kong, China (People’s Republic)

## Abstract

Non-Essentialism Belief of Aging (NEBA), the beliefs that the aging process is malleable, is closely related to subjective aging and motivation in old age. While previous studies have shown that NEBA predicts more positive subjective aging experiences, few studies have explored the underlying mechanisms. We propose that one crucial pathway might be attributional styles, the dispositional tendency to attribute negative health events to specific factors (e.g. lifestyle) or to general causes as old age. This study aims to examine the potential mediating effect of attribution styles on the relationship between NEBA and subjective aging, measured by awareness of age-related changes (AARC). N = 766 older adults aged 55-75 years (Mage = 61.8 ± 4.9 years) in China (N = 524), the United Kingdom (N = 122), and the United States (N = 120) responded to online survey instruments measuring NEBA, attribution styles, and AARC. Results showed that higher NEBA was associated with more specific attributional styles, was positively related to more AARC gains and fewer AARC losses across all three cultures. Moreover, age attribution styles fully mediated the relationship between NEBA and AARC gains and partially mediated the relationship between NEBA and AARC losses. Taken together, our findings reveal that attribution styles might be an important pathway through which NEBA influences subjective aging and well-being in old age.

